# Position Statement on Breastfeeding from the Italian Pediatric Societies

**DOI:** 10.1186/s13052-015-0191-x

**Published:** 2015-10-24

**Authors:** Riccardo Davanzo, Costantino Romagnoli, Giovanni Corsello

**Affiliations:** Division of Neonatology, Maternal and Child Health Institute-IRCCS “Burlo Garofolo” Trieste, Via dell’Istria 65/1, 34100 Trieste, Italy; Department of Pediatrics, Division of Neonatology, Catholic University S H, Rome, Italy; Department of Sciences for Health Promotion and Mother and Child Care, University of Palermo, Palermo, Italy

**Keywords:** Breastfeeding, Human milk, Position statement

## Abstract

The 2015 Position Statement on Breastfeeding of The Italian Pediatric Societies (SIP, SIN, SICupp, SIGENP) recognizes breastfeeding as an healthy behaviour with many short and long term benefits for both mother and infant.

While protecting, promoting and supporting breastfeeding, neonatologists and pediatricians need specific knowledge, skills and a positive attitude toward breastfeeding. In Maternity Hospitals and in Neonatal Units, appropriate organizative interventions should be applied in order to facilitate the beginning of breastfeeding and the use of mother’s/human milk.

The Italian Pediatric Societies indicate the desiderable goal of around 6 months exclusive breastfeeding if the infant grows properly according to WHO Growth Charts. In principle, complementary feeding should not be anticipated before 6 months as a nutritional strategy pretending to prevent allergy and/or celiac disease. Eventually, long term breastfeeding should be supported meeting mother’s desire.

## Background

Human milk is a individual-specific, biological and highly biodiverse liquid, that makes people equal in their nutrition and health opportunities. Moreover, breastfeeding, with its intimate relationship between mother and baby, is the cultural model for tolerance, variability-flexibility, and interaction between individuals and a worldwide action in terms of sustainability, environmental friendliness, and equality.

Limited data show that breastfeeding rates in Italy are suboptimal [1–4] when compared with Recommendations on Infant Feeding by WHO [5].

Given current epidemiological data on suboptimal Breastfeeding rates in Italy, health professionals, particularly pediatricians and neonatologists, have an important role in the protection, promotion and support of breastfeeding [6], both in Health Facilities and in the Comunity. Nevertheless, to effectively facilitate breastfeeding, they need specific knowledge and skills [7, 8] accompanied by a positive attitude toward breastfeeding [9].

As breastfeeding is a healthy behaviour, the Italian Pediatric Societies (Italian Society of Neonatology -SIN, Italian Society of Primary Pediatric Care -SICuPP, Italian Society of Pediatric Gastroenterology and Nutricion – SIGENP) have developed a statement to witness their position and to call pediatricians and neonatologists for action.

An *ad hoc* Working Group of the Italian Society of Pediatrics completed the Position Statement in August 2015. During September 2015 the Task Force on Breastfeeding of the Ministry of Health, Italy and the Executive Committees of SIP, SIN, SICupp and SIGENP approved the final version.

## Extract of the position statement on breastfeeding

The Position Statement on Breastfeeding (access to http://www.neonatologia.it/upload/2265_Position%20Statement%2013%20sett%202015.pdf for the full Italian text) can be resumed in the following points:

1. Breastfeeding carries well proven benefits to the health of both the mother and his/her baby [10, 11] and to the comunity in term of reduced costs for health care [12, 13] and to environmental sustainability
2. Breastfeeding should be considered as a nutritional norm. Although natural, breastfeeding must be learned by new mothers, who may face obstacles particularly at the beginning of breastfeeding, in the Maternity Hospitals as well as after hospital discharge. Breastfeeding mothers need advice and support from skilled professionals, who should keep a positive attitude toward breatfeeding.
3. While promoting breastfeeding, the neonatologist and the pediatrician should develop a general awareness on possible conflicts of interest, particularly regarding connection with baby food industry.
4. The informed choice of a mother for formula feeding should be accepted without discrimination versus a breastfeeding mother.
5. Data on breastfeeding rates should be collected using the WHO feeding definitions, both at Maternity and NICU discharge [14] and at least at the first immunization sessions at around 3 and 5 months of age.
6. The neonatologist and the pediatrician should develop an adequate knowledge and training on the physiology of lactation and the management of breastfeeding.
7. Breastfeeding should be contraindicated only for good medical reasons [15]. Radiological contrast agents are almost always compatible [16]. The lactation risk of medicines assumed by the mother should be assessed with an evidence based methodology, that ultimately demontrates that most drugs are safe while breastfeeding (9). Environmental contaminants should not be a reason to interrupt breastfeeding, as the benefits outweigh the risks. A new pregnancy in the first two trimesters is not a contraindication to breastfeed [17].
8. In Maternity Hospitals, neonatologist and pediatrician should use clinical and organizative protocols, that combine breastfeeding promotion with the evidence based neonatological and pediatric best practice [18, 19]
9. The Italian Pediatric Societies reccommend exclusive breastfeeding for about 6 months of life [20–22]. Poor growth properly assessed with the WHO Grow Charts [http://www.cdc.gov/growthcharts/who_charts.htm#The%20WHO%20Growth%20Charts] may suggest to anticipate the introduction of weaning foods between the fourth and the sixth month. Breastfeeding might continue after the introduction of semi-solids and solid foods, beyond 2 years of life according to mother ‘s desire [23–26].
10. Evidence based interventions proven to promote breastfeeding and the use of human milk should be applied in the NICUs [27]. Donor Milk Banks are component of this promotion [28, 29], although a cost/benefit ratio should be assessed before implementing a new one.

## Conclusion

The 2015 Position Statement of the Italian Pediatric Societies may represent a scientific and ethical reference tool for a more effective committment of neonatologists and pediatricians to increase breastfeeding among Italian pediatric population.
